# Modifying glycyrrhetinic acid liposomes with liver-targeting ligand of galactosylated derivative: preparation and evaluations

**DOI:** 10.18632/oncotarget.22143

**Published:** 2017-10-27

**Authors:** Jing Chen, Yuchao Chen, Yi Cheng, Youheng Gao, Pinjing Zheng, Chuangnan Li, Yidan Tong, Zhao Li, Wenhui Luo, Zhao Chen

**Affiliations:** ^1^ School of Chinese Materia Medica, Guangzhou University of Chinese Medicine, Guangdong, China; ^2^ The Second School of Clinic Medicine, Guangzhou University of Chinese Medicine, Guangdong, China; ^3^ Guangdong Second Traditional Chinese Medicine Hospital (Guangdong Research Institute of Traditional Chinese Medicine Engineering Technology), Guangdong, China

**Keywords:** liver-targeting, galactosylated derivative, glycyrrhetinic acid liposomes, liver disease therapeutic

## Abstract

In this study, novel glycyrrhetinic acid (GA) liposomes modified with a liver-targeting galactosylated derivative ligand (Gal) were prepared using a film-dispersion method. To characterize the samples, particle size, zeta potential, drug loading, and encapsulation efficiency were performed. Moreover, plasma and tissues were pre-treated by liquid-liquid extraction and analyzed by high-performance liquid chromatography-tandem mass spectrometry (LC-MS/MS). The results showed that the mean residence times (MRTs) and the area under the curve (AUC) of GA liposomes with Gal (Gal-GA-LP), and GA liposomes (GA-LP) were higher than the GA solution (GA-S) in plasma. The tissue (liver) distribution of Gal-GA-LP was significantly different in contrast to GA-LP. The relative intake rate (Re) of Gal-GA-LP and GA-LP in the liver was 4.752 and 2.196, respectively. The peak concentration ratio (Ce) of Gal-GA-LP and GA-LP in the liver was 2.796 and 1.083, respectively. The targeting efficiency (Te) of Gal-GA-LP and GA-LP in the liver was 48.193% and 34.718%, respectively. Taken together, the results indicate that Gal-GA-LP is an ideal complex for liver-targeting, and has great potential application in the clinical treatment of hepatic diseases. Drug loading and releasing experiments also indicated that most liposomes are spherical structures and have good dispersity under physiologic conditions, which could prolong GA release efficiency *in vitro*.

## INTRODUCTION

Glycyrrhetinic acid (GA), serving as an active principal ingredient of glycyrrhizin, is hydrolyzed by glucuronidase after oral administration [1]. GA has been identified as a potential anti-hepatotoxic agent [2, 3] and has been widely used in the clinical treatment of hepatic diseases [4, 5]. As an attractive inhibitor, GA has multi-beneficial pharmacologic activities, including anti-ulcerative [6], anti-inflammatory [7], anti-viral [8], and interferon induction [9]; however, GA is lipophilic and thus has low solubility in water (<0.01 mg/mL) and may cause unwanted sodium retention or potassium loss [10]. GA tablets are administered orally for most patients; injections are rarely reported. To avoid side effects and maintain the essential concentration of GA, drug delivery based on nanoparticles was developed. Nanoparticle and nanotechnologic materials, including liposomes, polymeric micelles, and dendrimers, have been shown to simultaneously have higher efficacy and lower cytotoxicity [11].

Liposomes are ideal formulations which have been approved by the Food and Drug Administration (FDA) [12]. A liposomal injection system is a highly credible method that increases the local concentration of a drug in the vicinity of tumors by altering the bio-distribution of associated drugs [13, 14]. Moreover, liposomes can reduce the amount of drug that penetrates healthy tissues, which is a major cause of cytotoxicity [15]. A targeting liposomal injection system is under development in our laboratory as a suitable strategy to increase the concentration of drugs in some specific tissue sites. The asialoglycoprotein receptor is abundant in hepatocytes, minimally expressed in extra-hepatic tissues, and provides attractive advantages for hepatocyte-mediated delivery [16]. Some reports have focused on glycosylated nanocarriers for targeted delivery to specific cell types have also been shown to be highly efficient [17, 18]. While hepatocytes display a number of targets, drug-ligand conjugates exhibit large variations based on different sites and ligands, thus galactosylated derivative ligands offer attractive options for asialoglycoprotein receptors [19, 20]. In our previous study we used a novel galactosylated derivative ligand (Gal) to enhance drug liver-targeting delivery. The novel Gal specifically recognizes the asialoglycoprotein receptor [21].

Herein, we determined whether or not the GA liposomes with Gal (Gal-GA-LP) have liver-targeting efficiency. First, we prepared GA liposomes (GA-LP) and Gal-GA-LP to improve GA anti-hepatic carcinoma and liver-targeting properties and characterized GA-LP and Gal-GA-LP by high-performance liquid chromatography-tandem mass spectrometry (LC-MS). Then, GA-LP and Gal-GA-LP were evaluated by release efficiency *in vitro*, hemolysis testing, and cellular uptake experiments. We also investigated the pharmacokinetics and targeting profile of Gal-GA-LP compared with GA-LP and GA solution (GA-S) by intravenous injection. The pharmacokinetic parameters included terminal elimination half-life (T_1/2z_), apparent tissue clearance (CL), apparent volume of distribution (V_d_), AUC, MRT, bio-distribution data, relative intake rate (Re), targeting efficiency (Te), and peak concentration ratio (Ce). Of note, the drug content of GA in plasma and tissues is in nanogram amounts only. Therefore, as a result, the high-performance liquid chromatography (HPLC) method is not suitable for pharmacokinetic and bio-distribution studies involving GA.

Compared with other detection techniques, the LC-MS/MS method is more sensitive, selective, and accurate. Some reports have studied GA in plasma by LC-MS/MS [22, 23], but simultaneous detection of GA in plasma and tissues has rarely been reported, and use of the LC-MS/MS method in detecting GA with GA liposomes and GA-S has never been reported. It was also has been reported that PEG-modified liposome loading GA, and a thorough review on GA receptor-targeting and GA-delivering carriers [24, 25]. However, this study was novel in that it provides a new kind of liposome modification that results in better cellular uptake of GA, as well as a LC/MS-based analysis of the liposomes.

It was the first time for our experimental team to use the synthesis of Gal compounds and modified glycyrrhetinic acid liposomes. Study the properties and target of liposomes *in vitro* and *in vivo*. I*n vitro* and *in vivo* evaluations are crucial steps before the clinical use of Gal-GA-LP. At the same time, LC-MS / MS method was used to detect the changes of GA in blood and tissue (heart, liver, spleen, lung and kidney). We anticipate that Gal-GA-LP will enhance liver-targeting efficiency and facilitate clinical treatment of liver disease.

## RESULTS

### GA-LP and Gal-GA-LP preparations

Encapsulation efficiency (EE), particle diameter, and polydispersity were measured as described above, and the results are shown in Table 1. When determining one factor, the other factors were regarded as fixed parameters. The first factor was the proportion of GA-to-blank liposomes. The results indicated that a suitable proportion of GA could improve the encapsulation efficiency, as well as dispersity; however, the particle size decreased gradually when improving the GA proportion because that GA could be mixed uniformly with blank liposomes only when relatively saturated. Thus, we determined the optimal proportion of 1:9 (GA/blank liposomes). The molar ratio of Gal: EPC, when serving as the second factor, was examined. We selected 5% as the molar ratio of Gal: EPC in subsequent tests. The type and amount of lyophilized protective agent were detected to form stable lyophilized GA liposomes without collapse. The preparations of the thin film dispersion and ethanol injection methods were compared, which showed that the former increased encapsulation efficiency and the latter decreased particle size and polydispersity index.

**Table 1:** Encapulation efficiency, mean particle diameter, and polydispersity index when one variable parameter was fixed

Order of evaluation	Variable	Level	Encapulation efficiency (%)±S.D.	Mean particle diameter (nm) ±S.D.	Polydispersityindex±S.D.
1st	The proportion of GA to blank liposomes (w/w)	1:6	81.45±0.60	225.43±0.65	0.5033±0.0071
		1:8	83.24±0.92	209.13±2.44	0.4230±0.0030
		1:9	91.06±0.59	221.17±2.76	0.4223±0.0055
		1:10	78.64±1.07	227.47±2.33	0.4830±0.0020
2nd	The molar ratio of Gal to EPC	3 %	91.28±0.67	190.60±0.75	0.3850±0.0046
		5 %	85.52±0.62	187.17±0.85	0.3217±0.0068
		7 %	84.55±0.10	196.17±0.86	0.4023±0.0068
		9 %	80.92±0.57	199.73±0.38	0.4410±0.0030
3rd	The type of cryoprotectant	mannitol	78.32±0.48	221.50±0.98	0.5383±0.0067
		trehalose	77.88±0.31	218.27±0.97	0.4530±0.0030
		sucrose	91.28±0.03	232.70±0.66	0.4793±0.0025
		glucose	86.94±0.20	189.33±0.75	0.5080±0.0026
		glucose-mannitol	93.22±0.62	185.83±0.47	0.3457±0.0025
		glucose-trehalose	87.25±0.75	212.37±0.96	0.4150±0.0025
		sucrose-trehalose	87.71±0.73	212.20±0.80	0.4280±0.0044
		sucrose-glucose	84.55±0.64	234.63±0.61	0.5347±0.0020
		mannitol-trehalose	88.65±0.39	225.20±0.75	0.4660±0.0034
		mannitol-sucrose	91.47±0.57	193.43±0.87	0.3853±0.0025
4th	The proportion of cryoprotectant to EPC (w/w)	4:1	82.57±0.66	225.30±1.28	0.4677±0.0051
		6:1	84.79±0.63	210.00±0.78	0.3413±0.0045
		8:1	81.83±0.84	218.27±0.65	0.4617±0.0045
		10:1	81.48±0.75	211.07±0.78	0.3997±0.0035
		12:1	81.03±0.99	230.37±0.82	0.4593±0.0056
5th	The method of preparation	thin-film dispersion method	92.77±0.62	181.00±0.66	0.3740±0.0045
		ethanol injection method	81.71±0.95	188.50±0.75	0.4983±0.0040

Notes: Data shown represent the mean ± S.D.

Abbreviations: S.D., standard deviation.

In brief, the optimal conditions for the production of GA-LP and Gal-GA-LP were as follows: the proportion of GA and blank liposomes was 1:9; the molar ratio of Gal: EPC was 5 %; the type of lyophilized protective agent was glucose-mannitol; the proportion of lyophilized protective agent: EPC was 6:1; and the thin film dispersion method was the method of preparation.

### Characterizations of GA-LP and Gal-GA-LP

**Figure 1:**
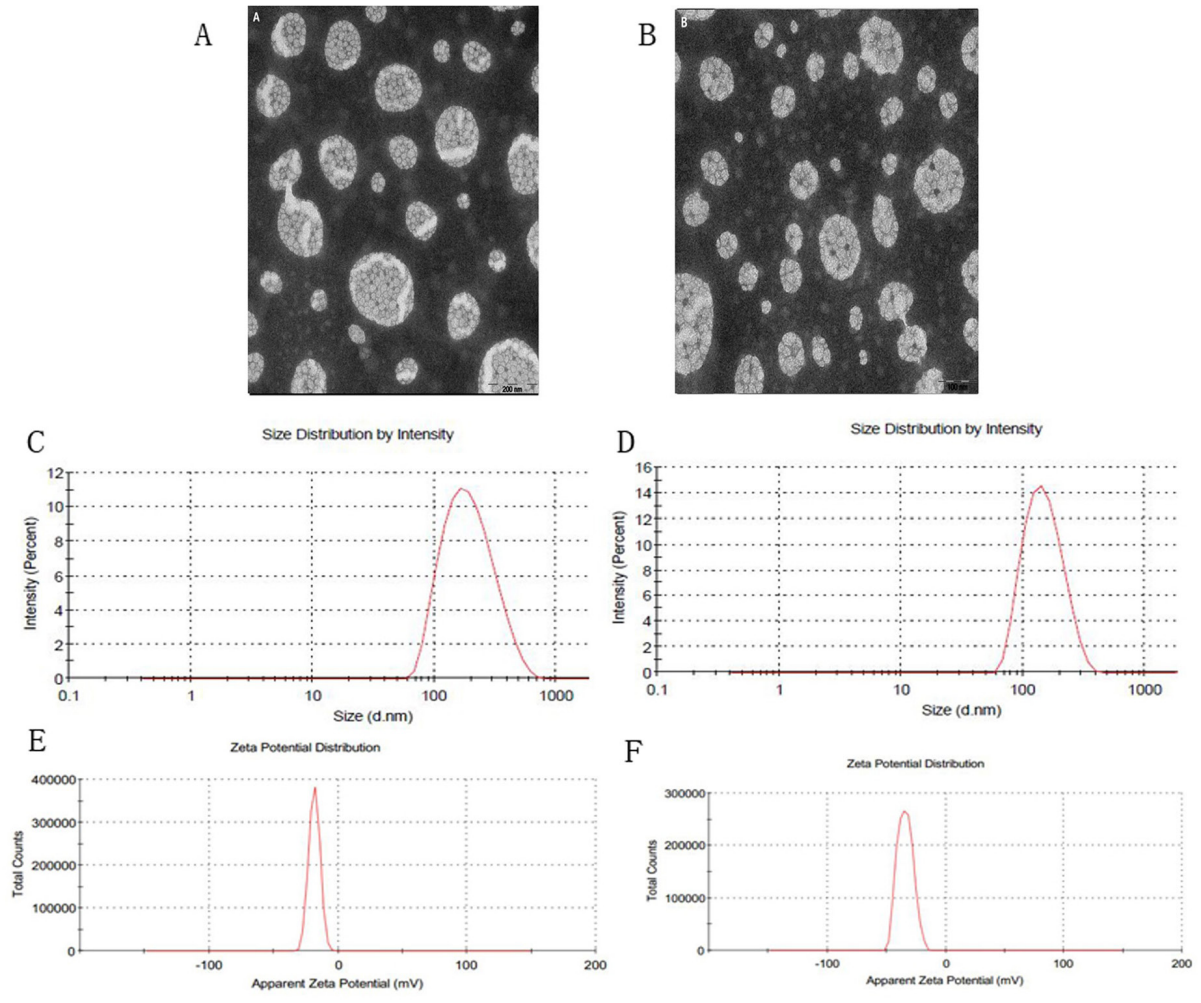
Transmission electron microscope photographs, size distributions and zeta potentials of GA-LP and Gal-GA-LP Note: Magnification ×50,000, **(A)** transmission electron microscope photographs of GA-LP, **(B)** transmission electron microscope photographs of Gal-GA-LP, **(C)** size distribution of GA-LP, **(D)** size distribution of Gal-GA-LP, **(E)** zeta potential of GA-LP, **(F)** zeta potential of Gal-GA-LP. Abbreviations: GA-LP, glycyrrhetinic acid liposomes; Gal-GA-LP, glycyrrhetinic acid liposomes modified with liver-targeting ligand of galactosylated derivative.

Spherical particles of GA-LP and Gal-GA-LP were observed in transmission electron microscope (TEM) images (Figure 1A, 1B). GA-LP and Gal-GA-LP were prepared using the thin film dispersion method under optimal conditions, and the results are shown in Table 2. The average sizes of Gal-GA-LP and GA-LP are shown in Figure 1C and 1D and the zeta potentials are shown in Figure 1E and 1F. The particle size of Gal-GA-LP was smaller than GA-LP. The Zetasizer Nano ZS90 Analyser showed that Gal-GA-LP had a similar size, suitable potential zeta, and uniform dispersion. In addition, the EE (%) and drug loading (DL) indicated the quality and clinical effects of the liposomes. The EE (%) values were > 80 % (92.89±0.91%) and the DL (%) values were good (8.97±0.09 %). No significant changes were observed when adding Gal to the liposome.

**Table 2:** The characterizations of the liposomes (n=3)

Sample	Particle size (nm) ±S.D.	Zeta potential (mV) ±S.D.	Polydispersity index±S.D.	Encapulation efficiency (%)±S.D.	Drug loading (%)±S.D.
GA-LP	192.10±2.30	-22.30±1.20	0.3923±0.0057	83.47±1.29	8.39±0.13
Gal-GA-LP	150.67±1.60	-35.50±0.96	0.2890±0.0062	92.89±0.91	8.97±0.09

Notes: Data shown represent the mean ± S.D.

Abbreviations: S.D., standard deviation; GA-LP, glycyrrhetinic acid liposomes; Gal-GA-LP, glycyrrhetinic acid liposomes modified with liver-targeting ligand of galactosylated derivative.

### Studies of *in vitro* drug release

**Figure 2:**
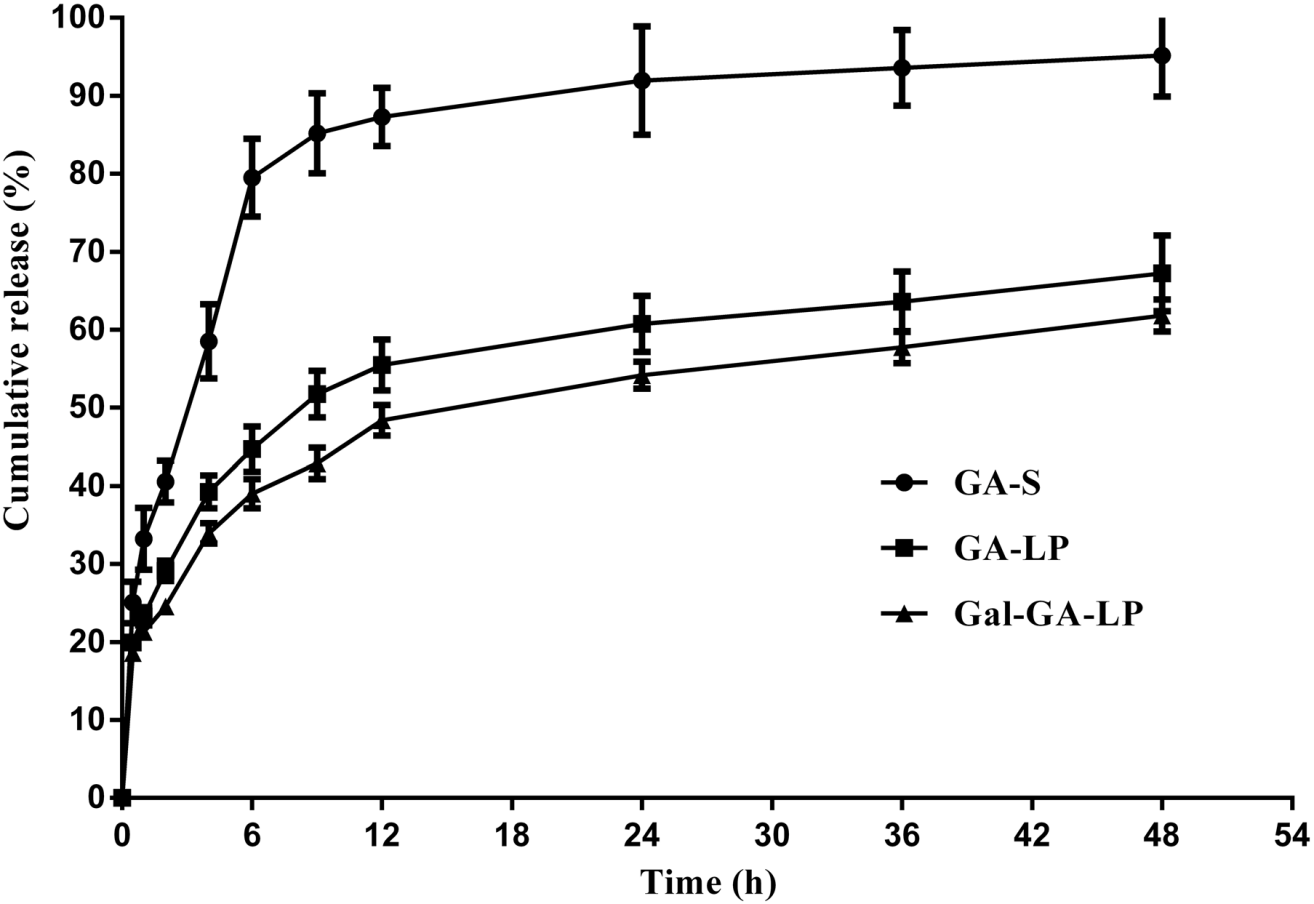
*In vitro* release of GA-S, GA-LP, and Gal-GA-LP Notes: (●) GA-S, (■) GA-LP, (▲) Gal-GA-LP, data shown represent the mean ± S.D. Abbreviations: GA-S, glycyrrhetinic acid solvent; GA-LP, glycyrrhetinic acid liposomes; Gal-GA-LP, glycyrrhetinic acid liposomes modified with liver-targeting ligand of galactosylated derivative.

The *in vitro* drug release behaviors of GA-S, GA-LP, and Gal-GA-LP have been used for comparison. The cumulative release behaviors of GA-S, GA-LP, and Gal-GA-LP are shown in Figure 2. Within 24 h at 37°C±0.5°C, > 90% of GA-S was released. In comparison, the GA-LP released 60% of drug, while Gal-GA-LP released 54%. Then, 40%-46% of the entrapped GA was further released during the subsequent 24 h of incubation. Taking into account that the release of GA from GA-S was rapid and almost complete within 12 h, the retention time of GA during release was dramatically prolonged by liposomal encapsulation. Moreover, no significant changes were found in terms of release characteristics when adding Gal to the liposomes.

### Stability

The stability data of GA-LP and Gal-GA-LP are summarized in Table 3. The leakage rate (LR) of GA-LP and Gal-GA-LP ranged from 1.53-6.08% and 1.41-5.45%, respectively. No aggregation or precipitation of nanoparticles was observed during storage for 3 months. The results showed that the properties of lyophilized GA-LP and Gal-GA-LP were stable to ensure > 94 % GA content in the liposomes. The stability study indicated that a suitable formulation (lyophilized liposomes) decreased the LR.

**Table 3:** The stability of GA liposomes (GA-LP and Gal-GA-LP)

Condition		Leakage rate (%)±S.D.	
		GA-LP	Gal-GA-LP
25°C±2°C, 60%±5% 2 weeks		2.39±0.49	2.00±0.20
25°C±2°C	1^st^month	1.53±0.54	1.41±0.55
	2^nd^ month	3.96±0.49	3.20±0.53
	3^rd^ month	6.08±0.62	5.45±0.41
4°C±1°C	3^rd^ month	3.28±0.27	2.75±0.73
	6^th^ month	4.53±0.87	4.51±0.90

Notes: Data shown represent the mean ± S.D.

Abbreviations: S.D., standard deviation; GA, glycyrrhetinic acid; GA-LP, glycyrrhetinic acid liposomes; Gal-GA-LP, glycyrrhetinic acid liposomes modified with liver-targeting ligand of galactosylated derivative.

### Hemolysis testing

**Figure 3:**
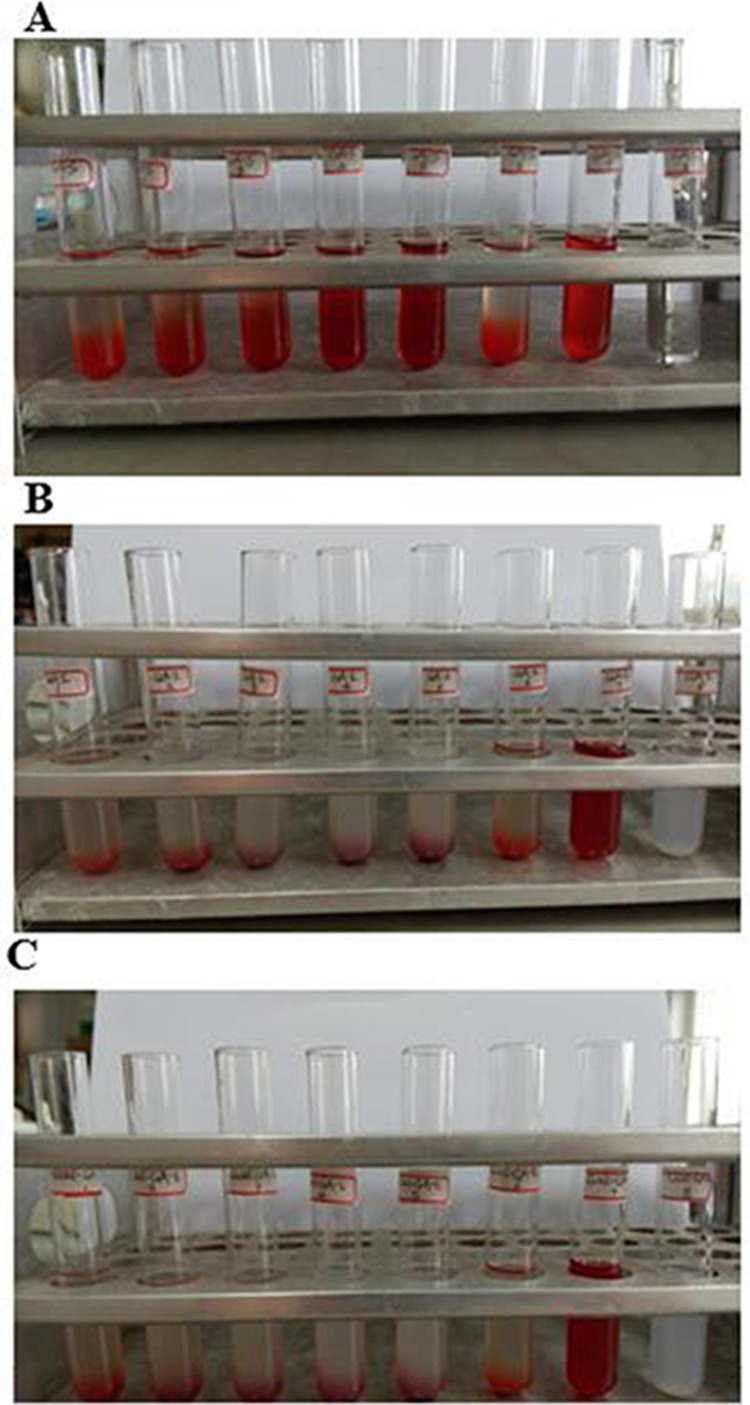
Hemolysis testing of GA-S, GA-LP, and Gal-GA-LP Notes: **(A)** GA-S, **(B)** GA-LP, **(C)** Gal-GA-LP. Abbreviations: GA-S, glycyrrhetinic acid solvent; GA-LP, glycyrrhetinic acid liposomes; Gal-GA-LP, glycyrrhetinic acid liposomes modified with liver-targeting ligand of galactosylated derivative.

Hemolysis data are shown in Figure 3. In two groups of GA liposomes, including GA-LP and Gal-GA-LP, hemolysis only occurred in the positive control tube for 6 h; however, the 4th and 5th GA-S tubes had the same phenomenon as the positive control tube with respect to hemolysis. After being re-mixed, red blood cells were uniformly distributed in tubes 1^st^-6^th^ of GA-LP and Gal-GA-LP without erythrocyte agglutination, while the 4th and 5^th^ GA-S tubes exhibited erythrocyte agglutination. The drug concentration of GA-LP and Gal-GA-LP was highest in the 5^th^ tube, in which there was no hemolysis and agglutination at 37°C. Therefore, the GA liposome is a safe formulation for injection. In addition, Gal is a safe drug carrier for targeted drug delivery.

### *In vitro* cellular uptake

**Figure 4:**
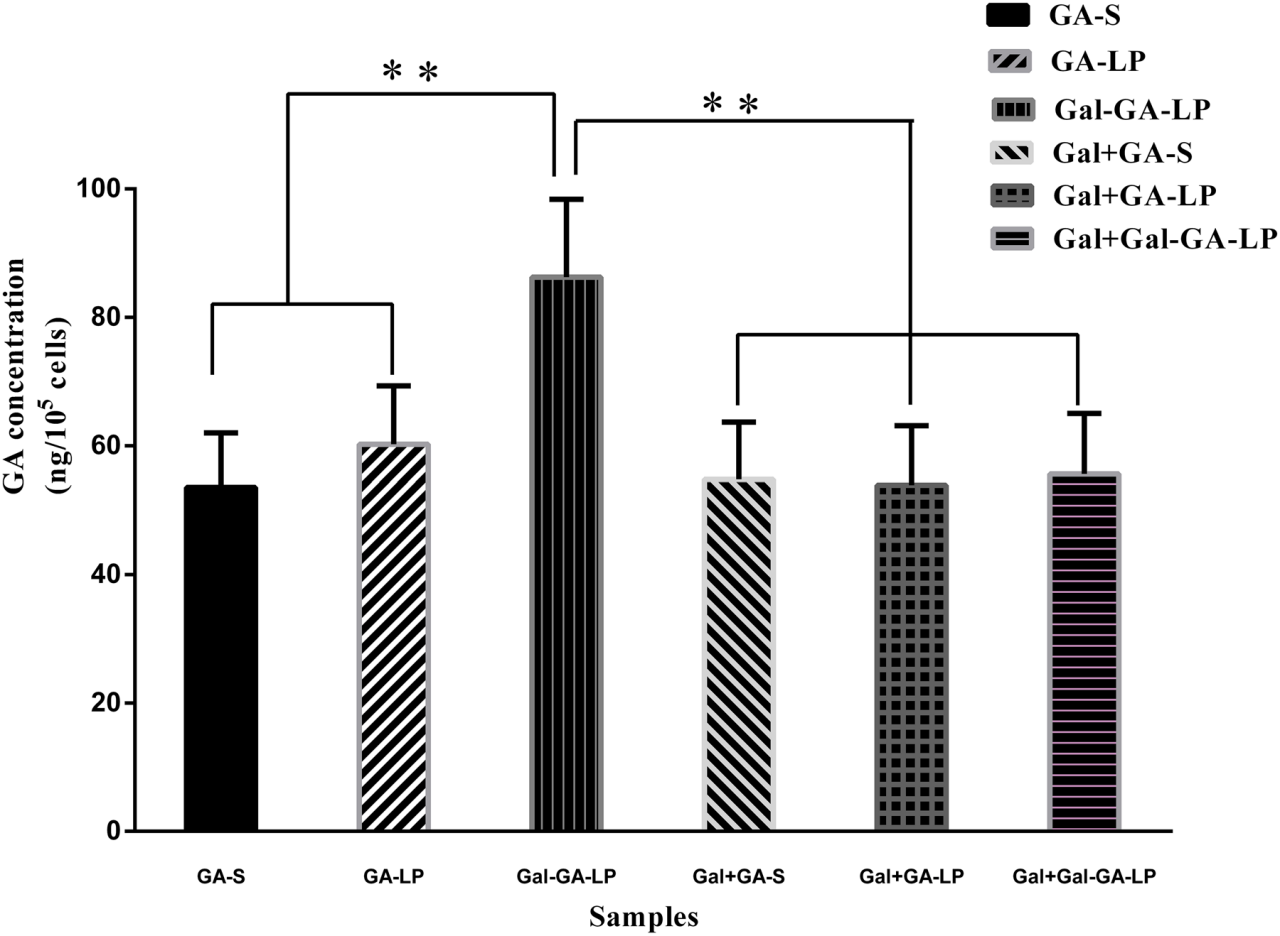
GA concentrations of GA-S, GA-LP, Gal-GA-LP, Gal+GA-S, Gal+GA-LP, and Gal+Gal-GA-LP in HepG2 cells Note: Data shown represent the mean ± S.D., ^**^P<0.05. Abbreviations: GA-S, glycyrrhetinic acid solvent; GA-LP, glycyrrhetinic acid liposomes; Gal-GA-LP, glycyrrhetinic acid liposomes modified with liver-targeting ligand of galactosylated derivative; Gal+GA-S, galactosylated derivative was added for 4h previously, then glycyrrhetinic acid solvent was added for further 6 h; Gal+GA-LP, galactosylated derivative was added for 4 h previously, then glycyrrhetinic acid liposomes were added for further 6 h; Gal+Gal-GA-LP, galactosylated derivative was added for 4 h previously, then glycyrrhetinic acid liposomes mediated with galactosylated derivative were added for further 6 h.

We determined the intracellular GA concentration using HPLC to determine whether or not the uptake of GA liposomes could be promoted in HepG2 cells. The results are shown in Figure 4. The amount of intracellular GA in GA-LP (60.24±9.07 ng/10^5^ cell) and Gal-GA-LP (86.28±12.13 ng/10^5^ cell) was greater than GA-S (53.59±8.44 ng/10^5^ cell), suggesting that GA in liposomes increased HepG2 cellular uptake, and Gal-GA-LP had a higher drug concentration than GA-LP because of Gal ligand. Therefore, we performed a competitive binding experiment with the addition of Gal beforehand, followed by GA-S, GA-LP, and Gal-GA-LP. As a result, the GA concentrations in HepG2 cells decreased significantly, indicating that Gal targeted the receptor in advance, and then hindered binding of Gal from Gal-GA-LP to the receptor. Thus, the GA concentration of Gal-GA-LP (1.57-fold) was greater than Gal+Gal-GA-LP.

### Optimization of LC-MS/MS conditions

### Chromatographic conditions

Ursolic acid was selected as the IS because its chromatographic behavior was similar to GA. To select the mobile-phase modifier, ammonium acetate was compared with acetic acid. Ammonium acetate not only enhanced deprotonation in the ESI-negative mode, but also obtained a good peak shape. An acetonitrile:5 mmoL ammonium acetate (70:30 [v/v]) water solution achieved excellent peak shape and obtained a maximum peak response. Satisfactory separation and a suitable retention time were achieved with a flow rate of 0.3 mL/min.

### Mass spectrometry

**Figure 5:**
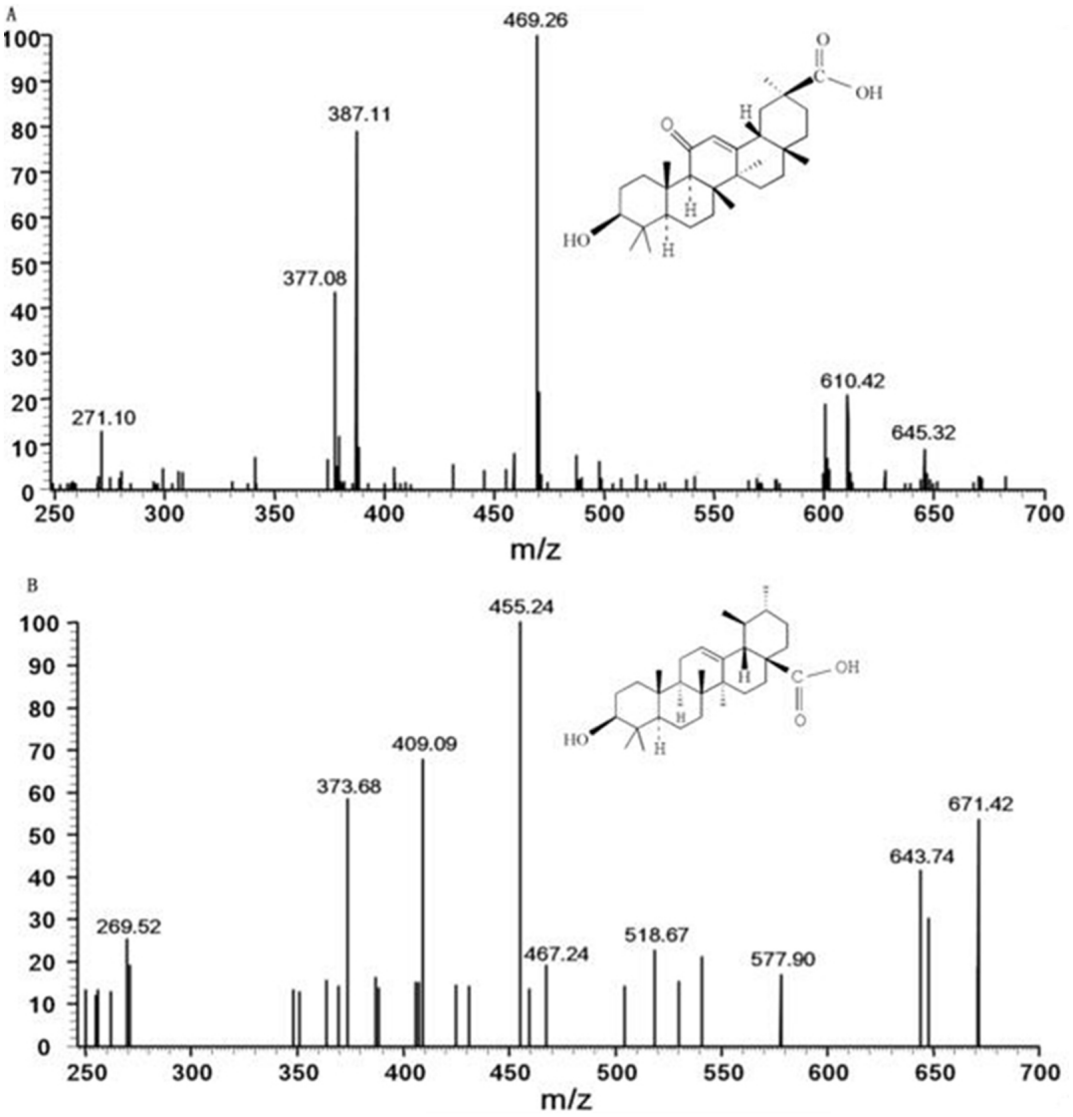
Scan spectra of production for GA and IS Notes: **(A)** GA. **(B)** IS Abbreviations: GA, glycyrrhetinic acid; IS, ursolic acid.

The signal transitions of GA and IS were observed after being broken into pieces in the collision cell. The signal from the m/z 469.26→387.11 transition was measured to be the most abundant and stable transition. Therefore, the transition of m/z 469.26→387.11 was selected for quantification of GA (Figure 5A). Similarly, the ion transition of m/z 455.24→409.09 was selected for IS (Figure 5B).

### Method validation

### Specificity and selectivity

**Figure 6:**
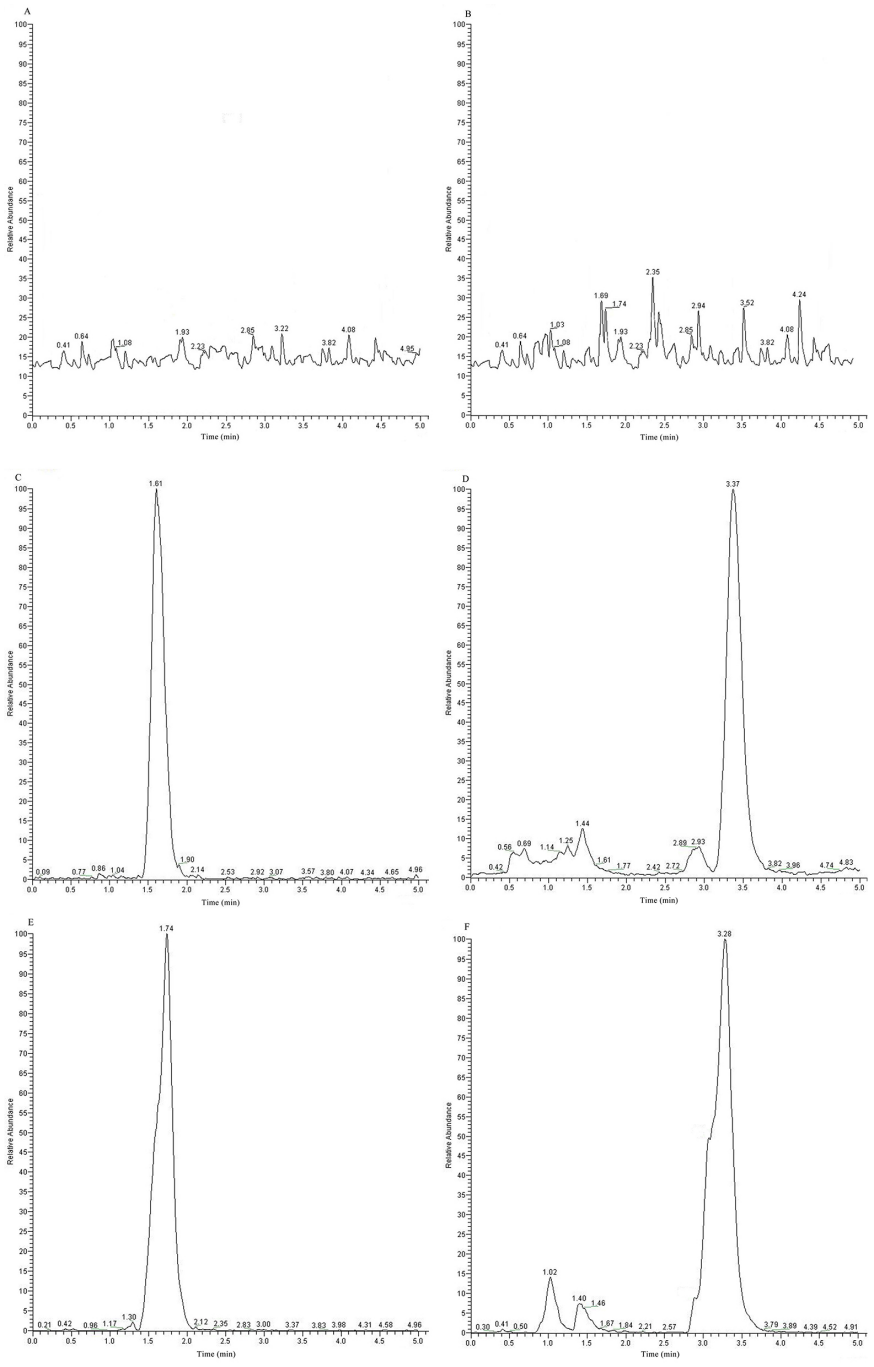
Representative LC-MS/MS chromatograms for GA and IS Notes: **(A)** A blank sample detected with GA channel. **(B)** A blank sample detected with IS channel. **(C)** A blank sample spiked with GA. **(D)** A blank sample spiked with IS. **(E)** A random sample after administration of GA. **(F)** A random sample after administration of GA spiked with IS Abbreviations: LC-MS/MS, high-performance liquid chromatography-tandem mass spectrometry; GA, glycyrrhetinic acid; IS, ursolic acid.

The newly developed simple method, LC-MS/MS, was more specific and less time consuming when compared with the previously reported methods [26, 27]. The typical LC-MS/MS chromatograms of blank plasma spiked with GA or IS, a random plasma or tissue homogenate sample after administration of GA, and a random sample spiked with IS are shown in Figure 6. The retention times of GA and IS were 1.61 and 3.37 min, respectively. Other LC-MS/MS chromatograms of bio-samples, including heart, liver, spleen, lung, and kidney, were similar to plasma (not shown in Figure 6). Thus, there was no significant endogenous interference in plasma and tissue homogenates of heart, liver, spleen, lung, and kidney, indicating that the method was selective.

### Linearity and limit of quantification

The linearity of the LC-MS/MS method was evaluated using a calibration curve within the range of 4–6000 ng/mL. The representative equations for the standard curves are listed in Table 4. In Table 4, Y represents the ratio of the GA peak area:IS and X represents the GA concentration. The LOQ denotes the lowest amount of analyte that could be precisely and accurately quantified. In the current study, the LOQ of plasma and various tissues was 3 ng/mL.

**Table 4:** Standard curves, linear coefficients and linear ranges of GA in different bio-samples

Bio-sample	Standard curve	Linear coefficient r^2^	Linear range (ng/mL)
Plasma of mice	Y=0.006X+0.059	0.9991	4-6000
Heart	Y=0.011X+0.182	0.9991	5-6000
Liver	Y=0.007X+0.217	0.9994	4-6000
Spleen	Y=0.005X+0.199	0.9991	4-6000
Lung	Y=0.007X+0.112	0.9994	4-6000
Kidney	Y=0.005X+0.158	0.9994	4-6000

Abbreviations: GA, glycyrrhetinic acid; Y, the peak area ratio of GA to IS; X, the concentration of GA.

### Precision and accuracy

The results of precision and accuracy are summarized in Table 5. As demonstrated, the intra- and inter-day precision (RSD) ranged from 2.63%-9.83% and 2.02%-8.19%, respectively. The accuracy (RE) of GA ranged from -3.71% to 9.60%. Both values were < 10%. Therefore, this analysis method had high accuracy and precision.

**Table 5:** Intra-day and Inter-day accuracy and precision of the method for determination of GA in biological samples

Biological sample	QC (ng/mL)	Intra-day (n=6)			Inter-day (n=6)		
		Mean concentration (ng/mL) ± S.D.	Accuracy (R.E.%)	Precision (R.S.D.%)	Mean concentration (ng/mL) ± S.D.	Accuracy (R.E.%)	Precision (R.S.D.%)
Plasma	25.00	25.19 ± 1.02	0.75	4.06	26.28 ± 1.10	5.13	4.17
	800.00	809.38 ± 24.21	1.17	2.99	822.24 ±16.57	2.78	2.02
	2000.00	1925.79 ± 139.23	-3.71	7.23	2119.71 ± 124.08	5.99	5.85
Heart	25.00	25.62 ± 1.01	2.48	3.96	26.68 ± 1.10	6.72	4.11
	800.00	791.45 ± 39.25	-1.07	4.96	838.11 ± 36.57	4.76	4.36
	2000.00	2051.49 ± 78.79	2.57	3.84	2190.51 ± 169.05	9.53	7.72
Liver	25.00	25.41 ± 1.28	1.63	5.04	26.01 ± 1.86	4.02	7.14
	800.00	825.17 ± 30.03	3.15	3.64	845.68 ± 31.87	5.71	3.77
	2000.00	1945.70 ± 70.76	-2.71	3.64	2191.93 ± 112.10	9.60	5.11
Spleen	25.00	25.47 ± 0.88	1.87	3.44	24.54 ± 1.56	-1.83	6.37
	800.00	779.13 ± 20.48	-2.61	2.63	832.60 ± 23.45	4.08	2.82
	2000.00	2126.29 ± 209.11	6.31	9.83	2189.88 ± 157.19	9.49	7.18
Lung	25.00	24.66 ± 1.04	-1.37	4.21	25.63 ± 1.73	2.53	6.76
	800.00	779.78 ± 26.37	-2.53	3.38	787.26 ± 48.25	-1.59	6.13
	2000.00	2049.59 ± 189.36	2.48	9.24	2142.81 ± 138.66	7.14	6.47
Kidney	25.00	25.85 ± 1.28	3.40	4.95	26.28 ± 1.16	5.11	4.41
	800.00	831.41 ± 28.78	3.93	3.46	853.46 ± 29.21	6.68	3.42
	2000.00	2070.00 ± 163.94	3.50	7.92	2178.93 ± 178.35	8.95	8.19

Note: R.S.D. (%) = (S.D./mean concentration) ^*^100 %; R.E. (%) = [(mean concentration/QC)-1] ^*^100 %.

Abbreviations: GA, glycyrrhetinic acid; QC, quality control samples; R.E., relative error; R.S.D., relative standard deviation; S.D., standard deviation.

### Recovery and matrix effect

Extraction recoveries and the matrix effect of GA are summarized in Table 6. The extraction recoveries of GA in plasma and tissues were in the ranges of 78.75%-84.75% and 79.37%-88.70%, respectively. In addition, the matrix effect of GA ranged from 87.98%-92.68%, 90.49%-92.96%, and 90.77%-94.01% at concentrations of 25, 800, and 2000 ng/mL, respectively. According to the requirements of the Pharmacopoeia of China People's Republic (2010 edition, part II), these extraction recoveries were within the acceptable range. The matrix effect indicated that the matrix had no significant matrix ionization suppression or enhancement.

**Table 6:** The extraction recovery and matrix effect of the method for determination of GA in biological samples (n=6)

Sample	QC (ng/mL)	Extraction recovery (%, mean ± S.D.)	R.S.D. (%)	Matrix effect (%, mean ± S.D.)	R.S.D. (%)
Plasma	25.00	84.33 ± 3.20	3.80	87.98 ± 4.56	5.19
	800.00	78.75 ± 3.37	4.28	91.43 ± 5.70	6.24
	2000.00	84.75 ± 2.32	2.74	93.67 ± 5.20	5.55
Heart	25.00	83.04 ± 5.25	6.32	90.30 ± 5.97	6.62
	800.00	84.31 ± 4.79	5.68	91.62 ± 4.40	4.81
	2000.00	88.70 ± 2.96	3.34	92.41 ± 4.37	4.73
Liver	25.00	85.45 ± 6.80	7.96	91.25 ± 6.47	7.09
	800.00	82.64 ± 5.15	6.23	92.56 ± 5.48	5.92
	2000.00	87.84 ± 6.52	7.42	93.19 ± 4.19	4.50
Spleen	25.00	82.25 ± 6.38	7.76	92.68 ± 3.32	3.59
	800.00	80.66 ± 6.73	8.34	91.85 ± 4.85	5.28
	2000.00	86.50 ± 3.47	4.02	90.77 ± 3.82	4.21
Lung	25.00	79.37 ± 7.64	9.63	91.76 ± 4.26	4.64
	800.00	84.22 ± 4.75	5.64	90.49 ± 2.91	3.22
	2000.00	87.45 ± 2.90	3.31	92.14 ± 3.94	4.28
Kidney	25.00	80.11 ± 6.10	7.61	91.91 ± 5.33	5.80
	800.00	81.93 ± 6.40	7.82	92.96 ± 3.99	4.30
	2000.00	86.41 ± 2.62	3.03	94.01 ± 3.86	4.11

Note: R.S.D. (%) = (S.D./mean concentration) ^*^100 %.

Abbreviations: GA, glycyrrhetinic acid; QC, quality control samples; R.S.D., relative standard deviation; S.D., standard deviation.

### Stability

The stability of GA in plasma and tissues (heart, liver, spleen, lung, and kidney) was determined at three concentrations (25, 800, and 2000 ng/mL). The results are shown in Table 7. Under the three conditions described above, the RSD was < 15 %. The results indicated that GA was stable under the experimental conditions.

**Table 7:** Stability of GA in plasma and tissue samples under different storage conditions (n = 6)

Storage condition	QC (ng/mL)	R.S.D. (%)					
		Plasma	Heart	Liver	Spleen	Lung	Kidney
Room temperature (25°C) for 24 h	25.00	5.58	3.50	5.30	5.40	6.07	6.71
	800.00	6.81	6.03	5.90	7.23	6.89	5.87
	2000.00	10.28	10.93	10.35	6.52	8.54	6.72
Frozen-thaw cycles at -20°C	25.00	5.73	5.28	4.66	4.01	6.17	5.20
	800.00	7.07	5.03	5.24	7.05	8.02	7.44
	2000.00	9.70	10.57	9.01	7.68	9.17	9.07
Frozen (-20°C) for 1 month	25.00	4.46	4.72	5.63	4.60	5.80	5.02
	800.00	5.10	4.23	9.10	5.06	4.73	4.82
	2000.00	8.87	12.70	6.41	9.40	7.88	8.17

Note: R.S.D. (%) = (S.D./mean concentration) ^*^100 %.

Abbreviations: GA, glycyrrhetinic acid; QC, quality control samples; R.S.D., relative standard deviation.

### Pharmacokinetic and bio-distribution study

**Figure 7:**
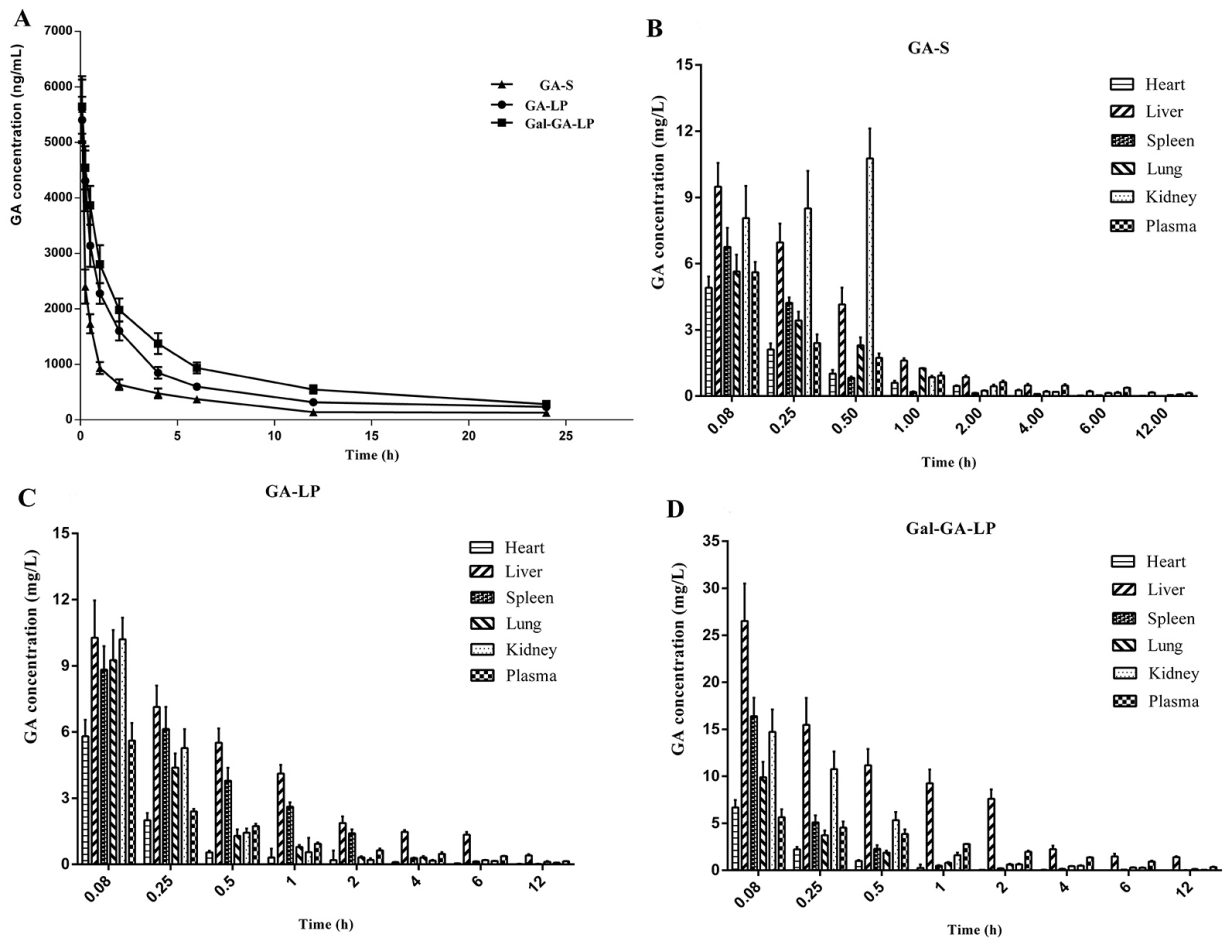
Mean plasma concentration of GA in mice after intravascular administration of GA-S, GA-LP, and Gal-GA-LP and distributions of GA in different organs at various time points after intravascular administration of GA-S, GA-LP, and Gal-GA-LP Note: Data shown represent the mean±S.D. Abbreviations: GA, glycyrrhetinic acid; GA-S, glycyrrhetinic acid solvent; GA-LP, glycyrrhetinic acid liposomes; Gal-GA-LP, glycyrrhetinic acid liposomes modified with liver-targeting ligand of galactosylated derivative.

The mean plasma concentration-time curves of GA-S, GA-LP, and Gal-GA-LP after a single intravenous injection are shown in Figure 7A. Compared with GA-S and GA-LP, the GA concentration from Gal-GA-LP declined slowly, which indicated that the GA liposome could prolong the active time because the formulation was removed slowly from the circulation [28]. The pharmacokinetic parameters, including elimination half-life (t_1/2z_), clearance (CL), volume of distribution (V_d_), area under the curve of drug concentration-time curve (AUC_0-∞_), and mean residence time (MRT_0-∞_), were analyzed using DAS 2.0 software. The main pharmacokinetic parameters are summarized in Table 8.

**Table 8:** Comparisons of plasma pharmacokinetic parameters of GA-S, GA-LP, and Gal-GA-LP after *i.v.* administration (mean ± S.D., n=5)

Parameters	GA-S	GA-LP	Gal-GA-LP
t_1/2z_ (h)	5.422 ± 2.632	5.819 ± 0.445^*^	7.487 ± 2.953^*^
CL (L h^-1^ kg^-1^)	1.929 ± 0.446	0.976 ± 0.099^*^	0.662 ± 0.059^*^
V_d_ (L kg^-1^)	13.953 ± 3.000	8.149 ± 0.280^*^	7.119 ± 2.869^*^
AUC_0-t_ (ug h L^-1^)	7373.510 ± 2243.468	15470.120 ± 1453.593^*^	21524.930 ± 2297.092^**^
AUC_0-∞_ (ug h L^-1^)	8493.645 ± 2238.985	16110.572 ± 1634.666^*^	23731.974 ± 2143.466^**^
MRT_0-t_ (h)	3.999 ± 1.786	6.248 ± 0.054^*^	6.616 ±0.980^*^
MRT_0-∞_ (h)	6.366 ± 2.628	7.276 ± 0.257^*^	9.440± 2.630^*^

Notes: Data shown represent the mean±S.D., ^*^, when compared with GA-S, *P*<0.05; ^**^, when compared with GA-S and GA-LP, *P*<0.05.

Abbreviations: GA-S, glycyrrhetinic acid solvent; GA-LP, glycyrrhetinic acid liposomes; Gal-GA-LP, glycyrrhetinic acid liposomes modified with liver-targeting ligand of galactosylated derivative; T_1/2_z, terminal elimination half-life; CL, apparent tissue clearance; Vd, apparent volume of distribution; AUC, area under concentration-time curve; MRT, mean residence time; S.D., standard deviation; *i.v.*, intravascular.

We confirmed whether or not the Gal-modified GA liposome had a liver-targeting efficiency *in vivo*. We studied the distributions of GA in heart, liver, spleen, lung, and kidney of mice at various time points after the intravenous administration of GA-S, GA-LP, and Gal-GA-LP. Then, we tested GA concentrations in tissue samples and the results are shown in Figure 7B-7D. The pharmacokinetic (AUC_0-∞_ and C_max_) and targeting parameters (Re, Te, and Ce) are summarized in Table 9. The GA concentration from Gal-GA-LP in the liver was clearly higher than other tissues.

**Table 9:** Pharmacokinetic parameters and targeting parameters of GA-S, GA-LP, Gal-GA-LP in plasma and various tissues of mice after i.v. administration (n=5)

Sample	Parameters	Heart	Liver	Spleen	Lung	Kidney	Plasma
GA-S	AUC (μg h L^-1^)	3471.481	9448.575	3217.687	4942.941	9819.824	7373.510
	C_max_ (μg L^-1^)	4907.228	9485.357	6760.000	5638.488	10764.400	5608.573
	Te (%)	9.070	24.687	8.407	12.915	25.657	19.265
GA-LP	AUC (μg h L^-1^)	2654.329	20746.629	9526.295	5918.741	5441.211	15470.120
	C_max_ (μg L^-1^)	5806.441	10269.646	8819.786	9253.503	10201.204	5403.583
	Re	0.765	2.196	2.961	1.197	0.554	2.098
	Te (%)	4.442	34.718	15.942	9.905	9.106	25.888
	Ce	1.183	1.083	1.305	1.641	0.948	0.963
Gal-GA-LP	AUC (μg h L^-1^)	2435.328	44901.029	6030.967	7074.443	11202.158	21524.930
	C_max_ (μg L^-1^)	2479.942	26525.004	16382.000	9888.715	14728.002	5643.000
	Re	0.702	4.752	1.874	1.431	1.141	2.919
	Te (%)	2.614	48.193	6.473	7.593	12.024	23.103
	Ce	0.505	2.796	2.423	1.754	1.368	1.006

Abbreviations: GA-S, glycyrrhetinic acid solvent; GA-LP, glycyrrhetinic acid liposomes; Gal-GA-LP, glycyrrhetinic acid liposomes modified with liver-targeting ligand of galactosylated derivative; AUC, area under concentration-time curve; Re, Relative intake rate; Te, Targeting efficiency; Ce, Peak concentration ratio; *i.v.*, intravascular.

## DISCUSSION

GA is an effective ingredient in treatment of hepatic dieases [4, 5]. However, GA is lipophilic and cause sodium retension or potassium loss [10]. Recent researchers in this area have focused on receptor-mediated drug delivery systems based on liposomes. The active targeting can be achieved through introduction of a targeting ligand into liposomes. Thus, a novel galactosylated derivates modified liposomes are designed for selective targeting of hepatic cells which are over-expressing asialoglycoprotein receptor.

In our previous study, Gal was synthesized successfully under the condition of lipase-catalytic. We chose the cholesterol because cholesterol was one of the components of liposomes, which could enhance stability the galactosyl moiety in liposomes. Lactitol containing galactosyl residue showed a higher affinity for asialoglycoprotein receptor. Divinyl sebacate was chosen as a spacer part of link between cholesterol and lactitol, the carbon chain length of bivinyl sebacate can meet the spatial distance between the galactosyl residues and the liposome surface.

The particle size plays an important role on drug distribution *in vivo*. It has been reported that the diameter of liposomes ranging from 100-200nm can significantly accumulate in tumor tissue on account of permeability and retention effect [29]. With an increasing amount of Gal, we reasoned that the liver-targeting phenomenon might be better while the encapsulation efficiency decreased and the particle size increased. Taking this into account, we selected 5% Gal. In our study, the particle size of GA-LP and Gal-GA-LP were less than 200 nm. We found that the higher the value of the zeta potential, the more difficult the electrostatic repulsion among these particles, which made the particles more stable in the dispersal system [30].

For the *in vitro* release study, the release of GA-S was relatively rapid while the release of GA-LP and Gal-GA-LP were slow. These data indicated that the incorporation of Gal into the liposome probably did not destroy the structure of the liposome. GA release was observed *in vitro* up to 48 h, and the data suggested that the application of liposomes would be increase the retention time of GA in the circulation and enhance the drug effects. The sustained release of GA revealed its applicability as a drug delivery system with continuous, slow release, and minimization of healthy tissue exposure when increasing the accumulation of therapeutic drugs in tumor sites.

The *in vitro* cellular uptake study was indicated that Gal-GA-LP might be transported into HepG2 cells by receptor-mediated endocytosis because of the Gal ligand, which specifically identified its receptor, and then exert its therapeutic effect after being released from the carrier. The cellular uptake results were in accordance with the results obtained by Guhagarkar et al [31–33]. The study showed that Gal-GA-LP is a promising targeting drug for hepatic diseases in the clinic.

The extraction methods of plasma and tissue samples were evaluated. The methods of protein precipitation (PPT), liquid-liquid extraction (LLE), and solid-phase extraction (SPE) were evaluated for the plasma and tissue preparations to establish a better method. PPT, including methanol, alcohol, and acetonitrile, failed to sufficiently remove endogenous interference, and SPE showed poor reproducibility due to the number of operating steps. The LLE method with various extracting solvents, including chloroform, ethyl acetate, and n-hexane, was investigated and evaluated for acceptable extraction recovery and matrix effect. Ethyl acetate with no concentration-dependent extraction recovery was adopted.

The data of pharmacokinetic parameters were showed the pharmacokinetic behaviors of GA in GA-S, GA-LP, and Gal-GA-LP. The elimination half-lives (t_1/2z_) of GA-LP (5.819 h) and Gal-GA-LP (7.487 h) were longer than GA-S (5.422 h). In addition, the mean plasma clearances (CL) for GA-LP (0.976 L h^-1^ kg^-1^) and Gal-GA-LP (0.662 L h^-1^ kg^-1^) were less than GA-S (1.929 L h^-1^ kg^-1^). These results indicated that GA in liposomes was cleared slowly from the blood. The relatively large values of the distribution volume (V_d_) of GA-LP (8.149 L kg^-1^) and Gal-GA-LP (7.119 L kg^-1^), when compared with GA-S (13.953 L kg^-1^), suggested that GA in liposomes could be easily distributed into tissues, which was beneficial to treat hepatic diseases. The areas under the curve of drug concentration (AUC_0-∞_) for GA-LP and Gal-GA-LP were 1.90-fold and 2.79-fold, respectively. These results indicated that Gal-GA-LP circulated longer in the blood than GA-S. Moreover, when compared with GA-S, higher mean residence times (MRT_0-∞_) of GA-LP (1.14-fold) and Gal-GA-LP (1.48-fold) indicated that GA liposomes prolong drug circulation in the blood [34].

Furthermore, the distributions study showed that GA concentration from Gal-GA-LP in the liver was clearly higher than other tissues, indicating that the Gal-modified liposome delivered GA mainly to the liver after the intravenous administration and demonstrated that GA liposomes modified with galactosylated lipid had enormous potential as a liver-targeting drug carrier [35, 36]. The AUC_0-∞_ of GA-LP (2.20-fold) and Gal-GA-LP (4.75-fold) was higher than GA-S in the liver, which suggested that Gal-modified GA liposomes contribute to enhance liver-targeting value. In addition, the target evaluations of three important parameters (Re, Te, and Ce) were calculated. Compared with GA-LP, the Re of Gal-GA-LP was the highest in the liver, which demonstrated that the release of GA to the liver was increased by Gal-modified liposomes. Compared with GA-S and GA-LP, Gal-GA-LP had outstanding liver-targeting with Te (48.193%) and Ce (2.796). The bio-distribution study indicated that Gal-GA-LP had better liver-targeting ability. These bio-distribution results were in agreement with those obtained by Biessen [37], which illustrated that galactosylated moieties are optimally recognized by liver. Thus, Gal-GA-LP could be enhanced for its bioavailability and targeted to the liver after intravenous injection.

In conclusion, GA liposomes modified with the liver-targeting ligand of Gal were prepared. The dynamic light scattering results showed that the spherical structure of Gal-GA-LP was approximately 170 nm and had good dispersion. Gal-GA-LP also exhibited high EE and low LR under physiologic conditions. Based on *in vitro* studies, the data demonstrated that the application of liposome formulation could prolong GA release and Gal was a safe drug carrier for targeted drug delivery to hepatocytes. In addition, the cellular uptake experiments illustrated that GA loaded with galactosylated moieties were ideal receptors and could be recognized by HepG2 cells. Furthermore, a LC-MS/MS method for quantifiable determination of GA in plasma and tissues was established successfully. Based on pharmacokinetic and bio-distribution studies, Gal-GA-LP showed better delivery efficiency and low cytotoxicity under intravenous injection. As a result, Gal-GA-LP may have huge potential as a promising nanocarrier in future clinical therapeutics.

## MATERIALS AND METHODS

### Materials

GA (assay 97.0%) was supplied by the China Resources Sanjiu Medical & Pharmaceutical (Shanghai, China). Ursolic acid (assay 99.2%, the internal standard [IS]) was purchased from the National Institutes for Food and Drug Control ([NIFDC], Guangzhou, China). Gal was synthesized by a non-aqueous enzymatic reaction in our laboratory. Egg phosphatidylcholine ([EPC], assay 98.0%) was purchased from Lipoid Co., Ltd. (Ludwigshafen, Germany). Cholesterol ([CH], assay 98%) was purchased from Advanced Vehicle Technology Pharmaceuticala. (Shanghai, China). Roswell Park Memorial Institute (RPMI)-1640 medium and trypsin solution (0.25%) were supplied by Thermo (Boston, MA, USA). Fetal bovine serum (FBS) was purchased from Hyclone (Logan, UT, USA). The hepatocellular carcinoma (HepG2) cell line was provided by the Institute of Biochemistry and Cell Biology, CAS (Shanghai, China). Methanol, ethanol, ammonium acetate, and ethyl acetate were purchased from Guangzhou Chemical Reagent Factory (Guangzhou, China) and were of at least analytical grade. HPLC or LC-MC/MS reagents were filtered through a 0.22μm filter before analysis. Methanol, ethanol, ethyl acetate, and the other reagents in liquid-liquid extraction were of analytical grade and used without further purification. Watsons’ water was used in all experiments.

#### GA-S

Sodium hydroxide (0.1 mol/L) was diluted with saline to adjust to a pH=8.2, then 7.8 mg of GA was dissolved in 5 mL of sodium hydroxide solution (pH=8.2) to prepare the desired concentration.

#### Stock solution

Stock solutions of GA and IS were prepared at 0.04 mg/mL by dissolving 2 mg in 50 mL of methanol, then stored at -20°C. Then, an amount of IS stock solution was diluted with methanol to make a concentration of 1 μg/mL and stored at -20°C. Phosphate buffered saline ([PBS], pH=7.4) was prepared with 0.136 g of monopotassium phosphate and 7.9 mL of 0.1 mol/L sodium hydroxide diluted in 100 mL of water, then filtered through a 0.22μm filter without particulate matter.

### Animals

Kunming mice (18–25 g; male and female mice in equal numbers) and New Zealand white rabbits (1.9-2.1 kg) were obtained from the Laboratory Animal Center of Guangzhou University of Chinese Medicine (Guangzhou, China). The animals were kept in cages under uniform experimental conditions (temperature, 25±2°C; humidity, 60±5%, dark-light cycle, 12 h). Food and water were freely available. The experiments involving the care and use of laboratory animals were conducted in strict accordance with National Institutes of Health guidelines [38] and the China National Institutes of Health.

### Preparation of modification of GA-LP and Gal-GA-LP

Gal-GA-LP was prepared using the thin-film dispersion method. In brief, EPC, CH, and GA (weight: 28.2, 8.2, and 4.18 mg, respectively) and Gal (5% of EPC, molar ratio) were dissolved in 5 mL of chloroform to form a mixed solution, then the organic solvent was removed under reduced pressure at 36–38°C by rotary evaporation to form a thin film on the inner walls of the round-bottomed flask. The vacuum was applied for 1 h to ensure total removal of any solvent trace. Glucose and mannitol (1:1, w/w) were dissolved in PBS (pH=7.4). The lipid film was then hydrated with 5 mL of PBS (pH=7.4) at 55°C by rotation (180r/min x 1 h) to form Gal-GA-LP. The injection of obtained liposomes was applied by probing ultrasonication for 10 min at 220W, then thrice-filtered through a polycarbonate filter with 0.22μm pores. To obtain a homogenous suspension, the injection was frozen at -20°C for 1 h, then dissolved in an ultrasonic water bath, repeated 3 times, then filtered with 0.22μm pores 3 times. The final liposomal vesicles were filled into penicillin bottles (10 mL), and pre-frozen at -80°C for 8 h. Gal-GA-LP suspensions were transferred to a freeze drier for 24 h to ensure the physical stability of the ultimate product. Lyophilized Gal-GA-LP powders were obtained. The procedure for producing conventional liposomes (GA-LP) without supplemental Gal were similar to Gal-GA-LP, but the weight of EPC, CH, and GA was 28.2, 9.4, and 4.2 mg, respectively.

In the current study, a single-factor method was used to determine optimal conditions. The proportion of GA to blank liposomes ranged from 1:10-1:6. The molar ratio of Gal: EPC ranged from 3%-9%. The types of cryoprotectant contained mannitol, trehalose, sucrose, glucose, glucose-mannitol, glucose-trehalose, sucrose-trehalose, sucrose-glucose, mannitol-trehalose, and mannitol-sucrose. The proportion of cryoprotectant and EPC ranged from 4:1-12:1 and a comparison between the ethanol injection and thin-film dispersion methods was made.

### Physicochemical characterizations of liposomes

#### Appearance

The GA-LP and Gal-GA-LP suspensions were semi-transparent white solutions with a visible sky-blue opalescence. The particle shapes were observed under a transmission electron microscope (TEM).

#### Particle size, polydispersity index, and distribution

A suitable amount of lyophilized GA-LP and Gal-GA-LP samples were dispersed in 5 mL of deionized water, and 3 mL of the solution was filled in a sample cell for detection purposes. The particle size, polydispersity index (PDI), and zeta-potential (ZP) of Gal-GA-LP and GA-LP were determined using a Zetasizer Nano ZS90 analyzer (Malvern Instruments, UK). Ensuring the reproducibility of experimental measuring conditions, the samples were adapted to the instrument systematically and automatically with a fixed angle of 90° to the incident light. The data were collected every 3 min.

#### Encapsulation efficiency (EE %) and drug loading (DL %)

Encapsulation efficiency (EE %) was expressed as the ratio of liposome-encapsulated drugs (E_drug_) to the total amount of drug (T_drug_) in the liposome preparation, and was measured using the Sephadex-50 filtration method. Drug loading (DL %) was expressed as the percentage of liposome-encapsulated drugs to the total amount of liposome-containing drug. EE % and DL % were calculated following Equations 1 and 2:

$$\text{EE}\%={\text{E}}_{\text{drug}}/{\text{T}}_{\text{drug}}\times 100\%$$ Eq.1

$$\text{DL}\%={\text{E}}_{\text{drug}}/{(\text{T}}_{\text{drug}}+{\text{T}}_{\text{liposomes}})\times 100\%$$ Eq.2

where E_drug_ is the weight of drug being encapsulated in the liposomes, T_drug_ is the weight of the total amount of charged drug, and T_liposomes_ is the weight of the total amount of blank liposomes.

To remove unentrapped drug completely, E_drug_ was evaluated after collecting the incorporated liposomes from 0.5 mL of the liposome suspension through a Sephadex G-50 mini-column (1.3×22 cm). Ten milliliters of the water fraction containing the liposomes of encapsulated drug was collected. To calculate the E_drug_, 0.2mL of the collected encapsulated drug fraction was disrupted by the addition of 0.8mL of methanol to form a clear solution. Similarly, to determine the T_drug_ in the liposome suspension, 0.9mL of methanol was added to 0.1mL of the GA liposome suspension to form a limpid solution. The concentrations of E_drug_ and T_drug_ were measured by HPLC analysis.

For the quantitative determination of GA, a reverse HPLC system was applied containing P680 HPLC pumps, an ASI-100 autosampler (Dionex, Sunnyvale, CA, USA), a UV detector (PDA-100 Photodiode Array; Dionex), and a BDS HYPERSIL C18 column (5 μm, 250×4.6 mm; Thermo Fisher Scientific Biological Chemical Co., Ltd.) The mobile phase was methanol-1 % acetic acid (89:11 [v/v]). The samples were determined at 254 nm by injecting a 10μL volume onto the column with a flow rate of 1 mL·min^-1^.

### *In vitro* drug release study

GA release from Gal-GA-LP, GA-LP, and GA-S was determined using the dialysis bag method. Lyophilized GA-LP, Gal-GA-LP powder, and GA-S containing 8 mg of GA were dissolved in 4 mL of PBS. Then, the solution was placed in dialysis bags (Thermo, USA). The bags were then put in 1000mL beakers containing 500 mL of PBS buffer (adjusting 0.1 mol to pH 7.4) under sinking conditions (100 rpm, 37°C±0.5°C). The release buffer (2.5 mL) was taken from the beaker at a pre-determined time (0.5, 1, 2, 4, 6, 9, 12, 24, 36, and 48 h) and refilled with the same amount of fresh medium at given time intervals. The supernatant (20 μL) was then injected directly into the HPLC system and the release of GA was analyzed. The released profiles were plotted. The accumulated release of GA-S, GA-LP, and Gal-GA-LP were calculated by the following formulas [39].

C1'=C1 Eq.3

Ci+1'=Ci+1−(V-Vi)∗Ci/V Eq.4

where C_*i*_ is the GA concentration of each sample (GA-S, GA-LP, and Gal-GA-LP) at pre-determined time intervals, C_*1*_′ is the increase of drug concentration during each time interval, V is the total volume of the released buffer, and V_*i*_ is the volume of each sample at pre-determined time intervals.

### Stability

Owing to the permeability of liposome membranes, the drug content was leaked from the liposome membrane within a period of time, which resulted in a decreasing encapsulation rate. The leakage rate (LR %) was a significant index to measure the stability of liposomes. According to the guidelines of the International Conference on Harmonisation [40], the stability studies of Gal-GA-LP, GA-LP, and GA-S were carried out under the following conditions: (1) temperature (25°C±2°C) and relative humidity (60%±5%) for 2 weeks; (2) temperature (25°C±2°C) for 1, 2, and 3 months; and (3) temperature (4°C±1°C) for 3 and 6 months. The stored samples were measured for the EE %. The LR (%) was calculated according to Equation 5:

$$\text{LR}(\%)=\left({\text{EE}}_{\text{i}}-{\text{EE}}_{\text{p}}\right)/{\text{EE}}_{\text{i}}\times 100\%$$ Eq.5

where EE_i_ is the encapsulation efficiency of the initial time and EE_p_ is the encapsulation efficiency of a pre-determined time.

### Hemolysis testing

This method of safe drug carriers has been reported [41]. The blood of New Zealand white rabbits was used to test the hemolysis effect of GA-S, GA-LP, and Gal-GA-LP. Blood samples (10 mL) were collected in heparinized test tubes and added to normal saline (10 mL), then vortexed for 1 min. The mixture was subsequently centrifuged at 1500 rpm for 15 min. The sedimented erythrocytes were collected, thrice-washed with saline (10 mL), and centrifuged repeatedly until the supernatant was no longer red [42]. Erythrocyte pellets (2 mL) were transferred to saline (98 mL) to prepare a 2 % erythrocyte standard suspension. GA-S, GA-LP, and Gal-GA-LP were dissolved in physiologic saline at a concentration of 4 mg/mL Twenty-four glass tubes were prepared and divided into three groups (GA-S, GA-LP, and Gal-GA-LP). The individuals of each group were numbered 1, 2, 3, 4, 5, 6, 7, and 8, as follows: tubes 1-5 (hemolysis of test samples); tube 6 (negative control); tube 7 (positive control); and tube 8 (reference of test sample [GA-S, GA-LP, or Gal-GA-LP]). The experimental design of hemolysis test was shown in Table 10. After blending, all the tubes were incubated at 37°C and observed at baseline after 6 h. Then, the suspension in each tube was remixed lightly after 24 h to observe agglutination of red cells.

**Table 10:** The experimental design of hemolysis test

Tube number	1	2	3	4	5	6	7	8
2 % erythrocyte standard suspension (mL)	2.5	2.5	2.5	2.5	2.5	2.5	2.5	0
Physiological saline (mL)	2.4	2.3	2.2	2.0	1.8	2.5	0	4.3
Distilled water (mL)	0	0	0	0	0	0	2.5	0
Sample (GA-S, GA-LP or Gal-GA-LP, mL)	0.1	0.2	0.3	0.5	0.7	0	0	0.7

Abbreviations: GA-S, glycyrrhetinic acid solvent; GA-LP, glycyrrhetinic acid liposomes; Gal-GA-LP, glycyrrhetinic acid liposomes modified with liver-targeting ligand of galactosylated derivative.

### *In vitro* cellular uptake

#### Cell culture

The HepG_2_ cell line was cultured in RPMI-1640 medium with 10% FBS and maintained at 37°C in an atmosphere containing 5% CO_2_. When the cells covered 75%–85% of the flask bottom, the cell medium was removed and the cells were thrice-flushed with PBS. The cells were detached from the flask by digestion with a 0.25% trypsin solution, and centrifuged at 1000 rpm for 10 min, then transferred to new flasks with fresh RPMI-1640 medium supplemented with 10% FBS.

#### Cellular uptake

HepG2 cells (1×10^5^cells/well) were cultured in 24-well plates with RPMI-1640 medium to determine cellular uptake. Cells were incubated with GA-S, GA-LP, and Gal-GA-LP containing 90 μmol/L of GA concentration for 6 h. After incubation, the cells were put in 500 μL of distilled water at -80°C three times, and then centrifuged at 12,000 r/min for 10 min (4°C). Ethyl acetate (1.5 mL) was added, vortexed for 5 min, and centrifuged again. The supernatant was evaporated in a vacuum oven at low temperature. The residue was reconstituted with 200 μL of methanol, vortex-mixed for 1 min, then centrifuged at 12 000 r/min for 10 min (4°C). Then, a 10μL aliquot of the sample was injected into a BDS HYPERSIL C_18_ column (5 μm, 250×4.6 mm). The composition of the mobile phase consisted of methanol-1 % acetic acid (89:11 [v/v]). The analysis was performed at a flow of 1 mL·min^-1^ by UV detector at 254 nm and 35°C. Finally, the concentration of GA was analyzed.

A competitive binding experiment was developed to evaluate the Gal receptor, which specifically mediated the cellular uptake of GA in Gal-GA-LP. HepG2 cells (1×10^5^cells/well) were seeded and exposed to 90 μmol/L Gal for 4 h, then incubated with GA-S, GA-LP, and Gal-GA-LP for 6 h. The subsequent process was the same as the above cellular uptake assay.

### Pharmacokinetics and bio-distribution studies

Animal experiments were performed according to the Guidelines of the Animal Center of Guangzhou University of Chinese Medicine. Animals were fed a standard laboratory diet with free access to water at a controlled temperature of 20-22°C and relative humidity of 65% with a 12 h light/dark cycle. Mice were kept fasting overnight with free access to water before experiments. One hundred thirty-five healthy Kunming mice (18±5 g) were randomly and evenly divided into three groups (GA-S, GA-LP, and Gal-GA-LP). Each group of mice was injected with a dose of 15.6 mg/kg via the caudal vein. Groups of five mice per liposome formulation per time point were used in this study. After injection, blood samples (0.50 mL) were obtained and collected directly into heparinized test tubes from the retro-orbital plexus at various times (0.08, 0.25, 0.5, 1, 2, 4, 6, 12, and 24 h). Mice were euthanized immediately, and the hearts, livers, spleens, lungs, and kidneys were collected. Plasma was separated by centrifugation (12,000 rpm for 10 min at 4°C), then stored at -20°C until use. Tissue samples were washed with ice cold physiologic saline, blotted, then wiped with filter paper, weighed, and homogenized in a 5-fold volume of normal saline (w/v). Homogenates were stored at -20°C until use. The following steps were performed in the preparation of plasma and tissue samples. The parameters were measured by a non-compartmental analysis using a DAS 2.0 computer program. According to the Pharmacopoeia of the People's Republic of China (2010 edition, part II), three distribution parameters (Re, Te, and Ce) for evaluation of liver targeting were measured [43].

The relative intake rate (Re) representing the target tissue was calculated as follows:

$$\text{Re}={\text{AUC}}_{\text{liposome}}/{\text{AUC}}_{\text{solution}}$$ Eq. 6

Targeting efficiency (Te), which indicates the target efficiency, was calculated as follows:

$$\text{Te}(\%)={\text{AUC}}_{\text{target}}/{\text{AUC}}_{\text{total}}{}^{*}100\%$$ Eq.7

The peak concentration ratio (Ce), which indicates the change in drug distribution, was calculated as follows:

$$\text{Ce}={\left({\text{C}}_{\max}\right)}_{\text{liposome}}/{\left({\text{C}}_{\max}\right)}_{{\text{solution}}^{.}}$$ Eq.8

### LC-MS/MS analysis of GA

#### Chromatographic conditions

The HPLC system consisted of 4-mode Surveyor LC pumps, a Surveyor autosampler, a CBM-20 system controller, and a Surveyor PDA. A BDS HYPERSIL C18 column (5 mm, 50×2.1 mm; Thermo) equipped with a 35°Ccolumn temperature was used for separation. The mobile phase consisted of acetonitrile and 5 mmoL ammonium acetate solvent (70:30 [v/v]). The solvent was filtered through a 0.22-μm filter and degassed. The ultraviolet (UV) absorption of GA in the samples was measured at 254 nm by injecting a 5μL volume into the column with a flow rate of 0.3 mL/min. The total run time was 5 min.

#### Mass spectrometric conditions

Mass spectrometer was conducted by a TSQ Quantum MS/MS system (Thermo). Following optimization of the settings, the instrument parameters were set at a nitrogen gas temperature of 300°C, spray voltage of 3000 V, sheath gas pressure of 30 psi, auxiliary gas pressure of 10 psi, and capillary temperature of 300 °C. The collision gas pressure for MS/MS was maintained at 34 V, and the scanning time was 5 min. Quantification was performed in selective reaction monitoring (SRM) to monitor the transition of m/z 469.26→387.11 for GA and m/z 455.24→409.09 for IS, respectively.

### Preparation of standard and quality control (QC) samples

GA (400 μg/mL) and IS (400 μg/mL) were made with methanol. Calibration standard solutions with concentrations of 4, 5, 10, 25, 50, 100, 200, 500, 1000, 2000, and 6000 ng/mL were made with methanol. The GA working solutions for QC samples at 3 different concentrations (25, 800, and 2000 ng/mL) were prepared in the same manner. The IS working solution (1000 ng/mL) was prepared in methanol from the IS stock solution. All the solutions were kept at -20°C and placed for approximately 5 min at room temperature before use.

A small amount of tissues was collected, weighed, and homogenized in normal saline (tissue–water ratio of 1:5 [w/v]). The plasma and tissue homogenates were used for the preparation of GA standards and QC samples. The 200 μL calibration standard solution at concentrations of 4, 5, 10, 25, 50, 100, 200, 500, 1000, 2000, and 6000 ng/mL was evaporated to dryness in a vacuum oven at low temperature, with addition of 200 μL of blank plasma or homogenates, 50 μL of IS working solution, and 1.3 mL of ethyl acetate, then vortex-mixed, centrifuged, and dried. The residue was added in 200 μL of acetonitrile to obtain concentrations of 4, 5, 10, 25, 50, 100, 200, 500, 1000, 2000, and 6000 ng/mL. The QC samples were prepared in the same manner at low, medium, and high GA concentrations (25, 800 and 2000 ng/mL, respectively).

### Plasma and tissue sample preparation

Plasma and tissue samples were treated by liquid-liquid extraction. Tissue samples (tissue: water=1:5 [w/v]) were homogenized with saline to form homogenates. Plasma or tissue homogenates (200 μL), 50 μL (1μg/mL) UA (IS), and 1.3 mL ethyl acetate were added to a 2 mL centrifuge tube. The mixture was vigorously vortex-mixed for 5 min, then centrifuged at 12 000 r/min for 10 min (4°C) to obtain a clean supernatant. The clean supernatant was transferred into a 2-mL clean test tube and evaporated to dryness in a vacuum oven at low temperature. The residue was reconstituted with 200 μL of acetonitrile, vortex-mixed for 1 min, then centrifuged at 12 000 r/min for 10 min (4°C). Then, a 5μL aliquot of the supernatant was injected into the LC-MS/MS system for analysis.

### Validation of the analytic method

The LC-MS/MS method was performed strictly in accordance with the FDA guidelines for validation of the bio-analytical method [44].

#### Specificity and selectivity

Specificity was investigated by analyzing samples (plasma or tissue homogenates) of a blank matrix with and without GA and IS. The selectivity of the method was assessed to determine the potential interference of endogenous compounds. The chromatograms of blank plasma or tissue homogenates were compared with blank plasma or tissue homogenates spiked with standard GA or IS, which is a random plasma or tissue homogenate sample after administration of GA and a random sample spiked with IS.

#### Linearity

Calibration curves consisting of various concentrations (4, 5, 10, 25, 50, 100, 200, 500, 1000, 2000, and 6000 ng/mL) were performed from working standard solutions of GA by plotting the concentrations of GA in plasma or tissues. The calibration curve was typically described by the equation Y = aX+b, where Y is the peak area ratio of GA:IS, and X is the concentration of GA. Each concentration sample was measured three times.

#### Limit of quantification (LOQ)

The limit of quantification (LOQ) was defined as the lowest concentration in the calibration curve that could be determined with sufficient precision < 20% and an accuracy within±20%. The LOQ was established in 5 continuous days for 6 replicates. The limit of detection (LOD) was defined as a signal-to-noise ratio of 3:1.

#### Precision and accuracy

The intra- and inter-day precision (expressed as the relative standard deviation [RSD]) and accuracy (expressed as the relative error [RE]) were determined. The intra-day precision and accuracy were evaluated by analyzing six replicates of the plasma and tissue homogenate samples at concentrations of 25, 800, and 2000 ng/mL. The inter-day precision and accuracy was measured by QC samples (plasma and tissue homogenate) over 6 continuous days.

#### Recovery and matrix effect (ME)

The extraction recoveries of GA and IS were calculated by comparing the peak area of pre-spiked samples with post-spiked samples by three concentrations for six replicates. The matrix effect was determined by measuring the corresponding peak ratio (analyte: IS) of the post-spiked samples. The same reference solution prepared in the mobile phase was used to measure the analyte peak ratio (anayte: IS) of GA: IS. Experiments were performed at concentration levels of 25, 800, and 2000 ng/mL for 6 replicates.

#### Stability

Sample stability was measured by analyzing the measured concentrations of low, medium, and high QC levels. The QC samples were evaluated under the following 3 storage conditions: (1) short-term stability at room temperature of 25°C for 24 h; (2) three frozen-thaw cycles at -20°C; and (3) long-term stability at -20°C for 1 month.

### Statistical analysis

The pharmacokinetic parameters, including T_1/2z_, CL, V_d_, AUC, MRT, and bio-distribution data, were calculated using a statistical moment algorithm (Drug and Statistics by DAS 2.0 program). Statistical significance of the differences among groups was determined using one-way analysis of variance (ANOVA), and single and multiple comparisons were performed using t-tests for independent groups to assume equal variance within each group. The results are presented as the mean ± standard deviation (S.D.). A *p*< 0.05 was set as the significance level for all tests.
